# Correction: The impact of believing you have had COVID-19 on self-reported behaviour: Cross-sectional survey

**DOI:** 10.1371/journal.pone.0248076

**Published:** 2021-02-25

**Authors:** Louise E. Smith, Abigail L. Mottershaw, Mark Egan, Jo Waller, Theresa M. Marteau, G. James Rubin

There are errors in Table 3. In the “Shopping for groceries/pharmacy” and “Shopping for items other than groceries/pharmacy” sections of the table, the values in the two columns under the heading “Self-reported behaviour n (%)” have been incorrectly switched. Please see the correct Table 3 here.

**Table 3:** Associations between thinking you have had COVID-19 and correct identification of most common symptoms of COVID-19; and adherence to social distancing measures (shopping for groceries/pharmacy, shopping for items other than groceries/pharmacy, and meeting up with friends and/or family who do not live with you; binary outcomes).

Thinks have had COVID-19?	Self-reported behaviour n (%)		Odds ratio (95% CI)	Adjusted odds ratio (95% CI)†
	**Shopping for groceries/pharmacy**			
	On one or fewer days in the last week n = 3760	On two or more days in the last week n = 2389		
No	2955 (63.5)	1701 (36.5)	Reference	Reference
Yes	805 (53.9)	688 (46.1)	0.67 (0.60 to 0.76)*	0.78 (0.69 to 0.89)*
	**Shopping for items other than groceries/pharmacy**			
	Not at all in the last week n = 4316	On one or more days in the last week n = 1833		
No	3500 (75.2)	1156 (24.8)	Reference	Reference
Yes	816 (54.7)	677 (45.3)	0.40 (0.35 to 0.45)*	0.51 (0.44 to 0.58)*
	**Meeting up with friends or family**			
	Not at all in the last week n = 5271	On one or more days in the last week n = 878		
No	4200 (90.2)	456 (9.8)	Reference	Reference
Yes	1071 (71.7)	422 (28.3)	0.28 (0.24 to 0.32)*	0.36 (0.30 to 0.43)*
	**Correct identification of cough and fever**			
	Did not correctly identify common symptoms n = 2390	Correctly identified common symptoms n = 3632		
No	1644 (36.0)	2927 (64.0)	Reference	Reference
Yes	746 (51.4)	705 (48.6)	0.53 (0.47 to 0.59)*	0.61 (0.54 to 0.69)*

*p≤.005

†Adjusting for all social and demographic characteristics and experimental condition
