# Supplementary material for: The impact of believing you have had COVID-19 on self-reported behaviour: Cross-sectional survey
Source: PLoS One. 2020 Nov 4;15(11):e0240399. doi: 10.1371/journal.pone.0240399 (PMC7641362; doi:10.1371/journal.pone.0240399)
Supplement: S1 File — (DOCX) [file pone.0240399.s001.docx]

**S1 File. Survey items**

1. **Overall, how worried are you about coronavirus? (SINGLE CODE)**

- Extremely worried
- Very worried
- Somewhat worried
- Not very worried
- Not at all worried

1. **To what extent do you think coronavirus poses a risk to: (SINGLE CODE PER STATEMENT)**

SCALE:

- Major risk
- Moderate risk
- Minor risk
- No risk at all

STATEMENTS:

- - To you personally?
  - People in the UK?

1. **Please enter the number of times you have been out of your home in the last seven days, for each of the following reasons? If you have not left your home for this reason, please write 0**

RANDOMISE ORDER

STATEMENTS

- To go to the shops, for groceries/pharmacy
- To go to the shops, for things other than groceries/pharmacy
- To go for a walk or some other exercise
- To go out to work
- To help or provide care for a vulnerable person
- To meet up with friends and/or family that you don’t live with

1. **Can you tell us what you think the most common symptoms of coronavirus are?**

**Please select up to 3 (MULTI CODE, RANDOMISE OPTIONS)**

- Cough
- High temperature / fever
- Shortness of breath / difficulty breathing
- Runny or blocked nose
- Aches and pains
- Chest pain
- Chills / shivering
- Sore throat
- Diarrhoea
- Headaches
- Stomach ache
- Feeling tired or having low energy
- Loss of sense of smell / taste
- Other, please state: (ALLOW TEXT INSERTION)
- Don’t know (SINGLE CODE)

1. **Do you know if you have had, or currently have, coronavirus? (SINGLE CODE)**

- I’ve definitely had it
- I think I’ve probably had it
- I think I’ve probably not had it
- I’ve definitely not had it

1. **Have you been tested for coronavirus? (SINGLE CODE)**

- Yes, the result showed I did have coronavirus
- Yes, the result showed I did not have coronavirus
- No, I haven’t been tested

1. **To what extent do you agree or disagree with the following statement. (SINGLE CODE)**

**I think I have some immunity to coronavirus**

- Strongly agree
- Agree
- Neither agree nor disagree
- Disagree
- Strongly disagree
